# Beyond genetics: including the environmental dimension in amyloidosis mouse models for Alzheimer’s disease

**DOI:** 10.1186/s40478-024-01747-6

**Published:** 2024-03-18

**Authors:** Lien Van Hoecke, Roosmarijn E. Vandenbroucke

**Affiliations:** 1 VIB-UGent Center for Inflammation Research, Technologiepark-Zwijnaarde 71, Ghent, 9052 Belgium; 2 Department of Biomedical Molecular Biology, Ghent University, Ghent, 9000 Belgium

The prevalence of Alzheimer’s disease (AD) continues to increase, with projections estimating 70 million patients by 2030. While recent FDA approvals for Aducanumab and Lecanemab have generated optimism, these therapies offer only partial relief by slowing cognitive decline [1–2], leaving us ill-equipped to face the looming AD epidemic. To better address AD, we must enhance our understanding of its pathophysiology, with a focus on utilizing animal models that closely replicate the human condition.

While mice are the most commonly used model to study neurodegenerative disorders, they don’t naturally exhibit AD-like pathology with age. The use of genetically engineered mice mimicking amyloidosis and/or tauopathy, two key pathological hallmarks of AD, has made substantial contribution to the field. When focusing on amyloidosis mouse models, these mice overexpress the amyloid β (Aβ) precursor protein (APP) along with various familial AD (FAD)-related mutations in APP or presenilin 1 (PS1), such as Tg2576, APP/PS1, 5xFAD and 3xTg-AD mice. However, these first-generation mouse models display characteristics unrelated to AD due to this (often cell type specific) APP overexpression. Second-generation knock-in mouse models, like *App*^*NL−G−F*^ mice, incorporated humanized sequences and clinical mutations within the mouse *App* gene, mitigating issues related to overexpression while still exhibiting Aβ accumulation. More recently, the field has seen the incorporation of xenografted human microglia [3–4] and neuron [4] AD mouse models, addressing the disparities between murine glial and neuronal cells and their human counterparts. These xenografted models create new opportunities to explore the functions of various genes identified in genetic studies, which may not be fully captured in conventional transgenic mouse models.

While the current Aβ deposition-based mouse models have significantly enriched our comprehension and contributed to therapy and biomarker development, their phenotype hinges exclusively on the presence of specific mutations responsible for the genetic variant of AD. This focus on genetic factors has led to an oversight of the increasing recognition of environmental factors as pivotal drivers and/or accelerators of AD development and progression.

## The environmental dimension of AD pathology

Environmental and lifestyle factors can promote peripheral inflammation, contributing to later cognitive decline [5–9]. Infections, even occurring years before dementia onset, elevate dementia risk through peripheral inflammatory and vascular pathways [10]. In addition, genome-wide association studies link immune response regulation genes, both within and outside the CNS, to increased risk of developing AD [11]. Aging, the most significant risk factor for AD, is accompanied by increase in pro-inflammatory markers in blood and tissues, or so called inflammaging [12]. Moreover, chronic diseases, such as obesity, diabetes and atherosclerosis are AD risk factors and are associated with a chronic pro-inflammatory state. Next to inflammatory events observed before the clinical onset of AD, patients often exhibit elevated levels of inflammatory markers compared to age-matched neurologically healthy individuals [13]. This comprehensive set of clinical findings underscores the role of environmental factors that trigger peripheral inflammation, and subsequently affect AD pathology. Preclinical studies have elucidated how peripheral inflammation affects AD pathology. First, peripheral inflammation activates the innate immune system leading to circulating pro-inflammatory cytokines impacting brain neurons and glial cells. In addition, peripheral immune cells can infiltrate in the brain, inducing glial activation and increased Aβ deposition. In the absence of inflammatory stimuli, microglia maintain a resting state, vigilantly monitoring their surroundings. However, prolonged activation disrupts their normal functions, leading to the release of cytokines and neurotoxic agents that contribute to neuronal cell death and impaired synaptic remodeling. Secondly, peripheral inflammation has shown to decrease Aβ clearance via impaired microglial Aβ phagocytosis and an LRP-1 dependent decreased Aβ transport from brain to blood in addition to increased Aβ influx into the brain. Thirdly, peripheral inflammation compromises brain barrier integrity, potentially allowing entry of blood-borne, often pro-inflammatory, cytokines and immune cells. Finally, emerging evidence links AD to intestinal inflammation and altered gut microbiota as this may lead to gut barrier dysfunction, allowing the release of pro-inflammatory molecules and gut microbes into the periphery.

Clearly, combined clinical and preclinical evidence emphasizes the association between peripheral inflammation and AD pathology. Environmental influences on AD require attention, prompting adjustments to mouse models considering these findings.

## Bridging the gap: incorporating environmental factors such as repetitive peripheral inflammation in AD mouse models

A significant portion of AD research is centred on mouse models within specific pathogen-free (SPF) environments. While this controlled environment reduces the experimental variability, it falls short in accurately replicating the environmental inflammatory conditions encountered by AD-inflicted patients. Additionally, the differences between SPF and conventionally housed mice, although often ignored or not reported in manuscripts, may contribute to variations in the reported onset timing of AD pathological hallmarks. As shown by preclinical studies discussed above, these peripheral inflammatory events affect crucial mechanisms behind the development of AD pathology. To bridge this disparity, it is vital to introduce a mechanism that triggers repetitive peripheral inflammation in mice without compromising the aforementioned experimental precision.

Peripheral infections and gut dysbiosis appear to primarily advance AD pathogenesis through general inflammation and vascular pathways, emphasizing the greater significance of inducing generalized peripheral inflammation in our AD mouse model, rather than focusing on any specific pathogen. Thereto, we previously introduced an AD mouse model [14] in which we twice administered lipopolysaccharide (LPS) to ${\sim}$ 5 months old *App*^*NL−G−F*^ mice to mimic multiple peripheral infection episodes or LPS leakage due to gut barrier dysfunction. This induction of repetitive peripheral inflammation led to increased Aβ deposition in the brain, heightened glial activation, and a more pronounced degree of neuronal dysfunction. This mouse model is used in our recent research article which show a significantly worsened AD pathology in *App*^NL−G−F^ mice that lack an adaptive immune system in the presence of repetitive peripheral inflammation [15]. Conversely, in the absence of peripheral triggers, we only observed a tendency toward reduced AD neuropathology. This further underscores the crucial role of systemic inflammation in the development of neuropathological characteristics and the importance of considering the impact of a too clean mouse environment, which does not accurately reflect the continuous immune challenges experienced in the human situation.

In conclusion, AD presents a multifaceted challenge that transcends genetic mutations. While mouse models exclusively based on genetic contributors have yielded valuable insights, it is essential not to underestimate the role of environmental factors, especially peripheral inflammation. To advance our understanding and develop effective treatments, a shift toward more comprehensive research models mirroring the full complexity of AD as it manifests in humans, is imperative.

## Data Availability

Not applicable.
